# IUC24405-80 Retrospective study of apalutamide/enzalutamide in hormone-sensitive metastatic prostate cancer

**DOI:** 10.1093/oncolo/oyaf248.030

**Published:** 2025-08-29

**Authors:** S Okinbaloye, G Olukiran, S Ramamurthy, A Fernandez-Ots, C Arias Quiroz, W Mmeka, A Zarien, K Taft, A Farrer, R Bebbington

**Affiliations:** Lincolnshire Community and Hospitals NHS Group, Lincoln, United Kingdom; Lincolnshire Community and Hospitals NHS Group, Lincoln, United Kingdom; Lincolnshire Community and Hospitals NHS Group, Lincoln, United Kingdom; Lincolnshire Community and Hospitals NHS Group, Lincoln, United Kingdom; Lincolnshire Community and Hospitals NHS Group, Lincoln, United Kingdom; Lincolnshire Community and Hospitals NHS Group, Lincoln, United Kingdom; Lincolnshire Community and Hospitals NHS Group, Lincoln, United Kingdom; Lincolnshire Community and Hospitals NHS Group, Lincoln, United Kingdom; Lincolnshire Community and Hospitals NHS Group, Lincoln, United Kingdom; Lincolnshire Community and Hospitals NHS Group, Lincoln, United Kingdom

## Abstract

**Background:**

Androgen receptor targeted agents (ARTA) with androgen deprivation treatment is the standard of care in hormone-sensitive metastatic prostate cancer (HSMPC). Retrospective study to compare the toxicities and tolerability of Enzalutamide and Apalutamide in HSMPC. To our knowledge, this is a novel comparison between the 2 drugs in real real-world setting.

**Methods:**

Data were collected from the chemotherapy registry, cancer drug fund website and clinic letters, between November 2020 and February 2022.

**Results:**

Seventy-three patients received Enzalutamide (E) and 58 received Apalutamide (A), in first line for HSMPC. Median follow-up period for the E group and A group was 128 and 102 weeks, respectively. The median patient age was 75 years in the E group and 74 years in the A group. The median prostate specific antigen (PSA) at the time of diagnosis was 571 ng/l in the E group and 293 ng/l in the A group. Post-treatment PSA was undetectable in 48% and 37% of the E and A groups, respectively. The commonest side effect is fatigue, noted in 48% of Patients in the E group and 33% of the A group. There were no reports of rash in the E group, while 16% of the A group reported varying degrees of skin rash. Hypothyroidism was seen in only the A group (16%). One patient in group E and 4 patients in group A had grade 3 hypertension. There were no incidences of cardiac failure in either. 21% and 7% in the A and E group, respectively, needed a break due to toxicities. 68.4% of the patient population still remain on Enzalutamide, while 63% of the patients remain on Apalutamide.

**Conclusions:**

In this retrospective analysis of a single-center analysis, we observed that rash and underactive thyroid were seen exclusively in the Apalutamide group. More numbers of patients on Apalutamide had breaks due to toxicities compared to Enzalutamide. The commonest side effect in both groups is fatigue.

